# Development and validation of a novel molecular biomarker diagnostic test for the early detection of sepsis

**DOI:** 10.1186/cc10274

**Published:** 2011-06-20

**Authors:** Allison Sutherland, Mervyn Thomas, Roslyn A Brandon, Richard B Brandon, Jeffrey Lipman, Benjamin Tang, Anthony McLean, Ranald Pascoe, Gareth Price, Thu Nguyen, Glenn Stone, Deon Venter

**Affiliations:** 1 Division of Immunobiology and Bioinformatics, Athlomics Pty Ltd, Jephson Street, Toowong, QLD 4066, Australia; 2 Burns Trauma and Critical Care Research Centre, The University of Queensland, Brisbane, Queensland, QLD 4072, Australia; 3 Department of Intensive Care Medicine, Royal Brisbane & Women's Hospital, Butterfield Street, Herston, QLD 4029, Australia; 4 Department of Intensive Care Medicine, Nepean Hospital, Western Clinical School, University of Sydney, Derby Street, Kingswood, NSW 2747, Australia; 5 Department of Intensive Care Medicine, Wesley Hospital, Coronation Drive, Auchenflower, QLD 4066, Australia; 6 Department of Pathology, Mater Health Services, Raymond Terrace, South Brisbane, QLD 4101, Australia

## Abstract

**Introduction:**

Sepsis is a complex immunological response to infection characterized by early hyper-inflammation followed by severe and protracted immunosuppression, suggesting that a multi-marker approach has the greatest clinical utility for early detection, within a clinical environment focused on Systemic Inflammatory Response Syndrome (SIRS) differentiation. Pre-clinical research using an equine sepsis model identified a panel of gene expression biomarkers that define the early aberrant immune activation. Thus, the primary objective was to apply these gene expression biomarkers to distinguish patients with sepsis from those who had undergone major open surgery and had clinical outcomes consistent with systemic inflammation due to physical trauma and wound healing.

**Methods:**

This was a multi-centre, prospective clinical trial conducted across four tertiary critical care settings in Australia. Sepsis patients were recruited if they met the 1992 Consensus Statement criteria and had clinical evidence of systemic infection based on microbiology diagnoses (*n *= 27). Participants in the post-surgical (PS) group were recruited pre-operatively and blood samples collected within 24 hours following surgery (*n *= 38). Healthy controls (HC) included hospital staff with no known concurrent illnesses (*n *= 20). Each participant had minimally 5 ml of PAXgene blood collected for leucocyte RNA isolation and gene expression analyses. Affymetrix array and multiplex tandem (MT)-PCR studies were conducted to evaluate transcriptional profiles in circulating white blood cells applying a set of 42 molecular markers that had been identified *a priori*. A LogitBoost algorithm was used to create a machine learning diagnostic rule to predict sepsis outcomes.

**Results:**

Based on preliminary microarray analyses comparing HC and sepsis groups, a panel of 42-gene expression markers were identified that represented key innate and adaptive immune function, cell cycling, WBC differentiation, extracellular remodelling and immune modulation pathways. Comparisons against GEO data confirmed the definitive separation of the sepsis cohort. Quantitative PCR results suggest the capacity for this test to differentiate severe systemic inflammation from HC is 92%. The area under the curve (AUC) receiver operator characteristics (ROC) curve findings demonstrated sepsis prediction within a mixed inflammatory population, was between 86 and 92%.

**Conclusions:**

This novel molecular biomarker test has a clinically relevant sensitivity and specificity profile, and has the capacity for early detection of sepsis via the monitoring of critical care patients.

## Introduction

Systemic Inflammatory Response Syndrome (SIRS) is an overwhelming whole body reaction that may have an infectious or non-infectious aetiology and is described in association with critical conditions that include, but are not limited to, pancreatitis, ischemia, trauma and severe tissue injury, including that encountered in patients having major surgery. Major open surgery is a controlled form of physical insult that results in varying degrees of systemic inflammation. To date, it has been reported that the occurrence of SIRS following cardiac bypass surgery [1,2], is common, as well as being a major cause of postoperative complications including death. In particular, Michalopoulos and colleagues observed that within the immediate 12 hours following Coronary Artery Bypass Surgery, 100% of their study population (*n *= 2,615) had clinical signs and symptoms consistent with SIRS. This SIRS response is also known to occur post-operatively following other types of major procedures [3,4]; however, only in cardiac surgical patients has this uniformity of response been demonstrated in the intensive care setting.

If infection is suspected in addition to SIRS presentation, the term sepsis is applied [5,6]. This condition is the leading cause of mortality in the adult intensive care unit (ICU), ranging from between 18 to 50% [7-9]. In developed countries, the incidence of sepsis is expected to rise due to aging populations, immune-compromised patients, increasing longevity of patients with chronic diseases, antimicrobial resistance, especially in younger people, as well as viral illnesses such as AIDS.

Recent research has suggested that, owing to the amount of cellular necrosis from major physical injury and trauma, mitochondrial DNA is released into the circulation, where it is capable of eliciting inflammatory signals, also referred to as damage (or danger)-associated molecular patterns (DAMPs) [10]. It is these DAMPS (which are also contributed to by nuclear DNA, purine metabolites, as well as other nuclear and cytosolic proteins), that stimulate an acute phase response by the innate immune system which is biologically concordant with pathogen-associated molecular patterns released during infection. Furthermore, the presence of DNA anywhere other than the nucleus or mitochondrial nucleoids elicits a response mediated by toll-like receptor-9 and DNA-dependent activator of interferon regulatory factors (DAI), resulting in the activation of the innate immune response, and can also be associated with the generation of autoantibodies and immune complexes [11]. Thus, following major physical trauma or other pathophysiological processes that result in cell lysis *en masse*, the immune system reacts in a similar manner to that which occurs when sterile tissues become infected. Moreover, the findings from Zhang *et al*. [10], demonstrate the difficulty in separating infectious from non-infectious SIRS when the innate immune system responds similarly to both conditions.

For many decades, the cornerstone of sepsis diagnosis and treatment has been identifying the causative circulating pathogen and quantitating single immune-related blood analytes -- medical determinants which are not necessarily specific to sepsis, but routinely evaluated to assess the patient's physiological response to the pathogen. In contrast, it has recently been proposed by Hotchkiss *et al*. [12], that the sepsis condition is multifactorial and inclusive of the both an early exuberant innate immune response (or "hyperinflammation") followed by a stage of protracted immunosuppression that is referred to as immunoparalysis [12-14]. While this is still conjecture, there would appear to be a combination of phases involved in septic episodes that are not necessarily assessable in terms of presentation, physiology, chemistry, or pathogen load. Given that the immune response to sepsis is complex and difficult to evaluate with single analytes, high throughput technologies such as multiplex PCR have substantive utility for the clinical development of a diagnostic test that is capable of evaluating perturbations in circulating gene expression profiles, and thus determining the status of the patient's immune system.

Thus, as the majority of patients admitted to the tertiary care ICU setting have undifferentiated SIRS, it is of great clinical importance that those patients who have a suspected infection or are at high risk of infection can be identified early, in order to initiate evidence-based and goal-orientated medical therapy. Hence, the primary objective was to validate a molecular biomarker signature identified *a priori *from pre-clinical research by determining performance outcomes, in a population of critical care patients that included post-operative surgical patients and blood culture-positive sepsis patient.

## Materials and methods

### Study design and research governance

This was a multi-centre, prospective, observational clinical trial conducted across four tertiary critical care settings in Australia from November 2007 to November 2009. Athlomics Pty Ltd sponsored the trial and has registered this product as the SeptiCyte^® ^Lab test. The sponsor initiated and designed the trial in collaboration with clinical investigators. The study protocol was approved by institutional review boards (IRBs)/Human Research Ethics Committees (HRECs) from Mater Health Services (MHS), Uniting Care, the Royal Brisbane & Women's Hospital and the Nepean Hospital Human Research Ethics Committee, prior to the recruitment of study volunteers. Independent clinical research organisations contracted by the sponsor were responsible for the monitoring and management of clinical data including verification with source notes.

Data collected from the aforementioned clinical trial were used to perform microarray studies in which to define a gene set in which to focus MT PCR studies. Following, an *a priori *panel of gene expression biomarkers was applied to MT PCR data from the clinical trial to create a diagnostic rule. The MT PCR data were randomly partitioned into a training set and a test set. The diagnostic rule was generated from the training set, and then applied to the test set, in a blinded fashion. MT and GS performed bioinformatics and statistical analyses in accordance with details presented in the study protocol and statistical analysis plan.

### Study population and criteria for inclusion and exclusion

All study participants were 18 years or older, had a body mass index < 40, and provided written informed consent. Patients were recruited as being likely to enter the Sepsis cohort if they met the ACCP/SCCM Consensus Statement [6], and had a clinical suspicion of systemic infection based on microbiological diagnoses. A definitive diagnosis of sepsis was unlikely to be known at the time patients were enrolled in the study; thus confirmation of sepsis and assignment of patients to the sepsis cohort was made retrospectively.

Potential sepsis participants admitted to the ICU and, patients admitted for planned major open surgery, were excluded from the study if they had any systemic immunological disorders including Systemic Lupus Erythromatosus, Crohn's disease and Insulin-Dependent Diabetes Mellitus (Type 1 diabetes); were transplant recipients or were currently receiving chemotherapy treatment for cancer.

Twenty-seven blood culture positive sepsis patients with community-acquired infections were enrolled into this clinical trial as soon as practicable, on admission to the ICU. Specifically, sepsis patients' were enrolled within 24 hours of admission and on average, were in the ICU for five days. Participants in the post-surgical (PS) cohort were recruited pre-operatively and blood samples were collected within 24 hours following surgery (*n *= 38). Furthermore, 20 healthy adult control (HC) participants were recruited within the Mater Adult Hospital staff, on the basis that they had no concurrent illnesses at the time of blood collection or any past history of immunological dysfunction. All participants or their surrogate decision-maker provided written informed consent prior to the collection of any study data or biological samples.

### Collection of data

Demography, vital signs measurements (blood pressure, heart rate, respiratory rate, tympanic temperature), haematology (full blood count), clinical chemistry (urea, electrolytes, liver function enzymes, blood glucose) as well as microbial status were recorded. Blood was drawn into minimally two PAXgene (PreAnalytix, Feldbachstrasse, Hombrechtikon, Switzerland) tubes (5 ml total) for gene expression analyses using the SeptiCyte Lab test.

### Gene expression assays

RNA isolation was performed using PAXgene Blood RNA kits (PreAnalytix, a Qiagen/BD Company, Feldbachstrasse, Switzerland) and following standard instructions recommended by the manufacturer. RNA quality was determined using an automated electrophoresis station (Experion, BioRad (Gladesville, New South Wales, Australia) and BioAnalyser, Agilent (Forest Hill, Victoria, Australia). The 260 nm/280 nm ratios for all samples were > 1.9. Once total RNA was extracted, gene expression was assessed using the Affymetrix HGU133 Plus 2.0 GeneChip^® ^(Santa Clara, CA, USA) and MT-PCR SeptiCyte Lab assay.

Microarray studies were conducted on total RNA extracted from a subset of participants that included 20 HC, 11 post-surgical and 10 sepsis samples, using HGU133 Plus 2.0 GeneChips (Affymetrix, Santa Clara, CA, USA). Of the 145 gene expression biomarkers derived from the early sepsis equine model, 408 probesets were derived. This panel was selected because it had been demonstrated *a priori *to contain information relevant to the separation of the three groups. In brief, 10 μg of total RNA was processed using the 3' Amplification One-Cycle Target Labelling kit (Affymetrix) and 20 μg of generated cRNA was injected into a GeneChip cartridge. The GeneChip array was incubated at 45°C for minimally 16 hr in a rotating oven at 60 rpm. GeneChips were then washed and stained with Streptavidin Phycoerythrin, using Affymetrix supplied wash protocol, EukGE-Ws2v5.

HGU133 fluorescent images were acquired in a GeneChip^® ^Scanner 3000 7G (Affymetrix). Affymetrix Power Tools, R and Perl scripts were used to filter background noise and normalise data based on a detection metric used to identify perfect match probes relative to other background probes. Differentially expressed genes were compared if the signal was > 100 and the fold change was > 2.0. All HGU133 Plus 2.0 GeneChip data are available from the GEO repository (GSE28750).

The MT-PCR approach first described by Stanley and Szewczuk [15] combines reverse transcription and preliminary amplification within Step 1 followed by final target amplification in Step 2. In brief, a mastermix was prepared that contained in part, lyophilized primers for Step 1 of this method and 10 ng of RNA template. Primer sets were designed by AusDiagnostics Pty Ltd (Sydney, NSW, Australia), and validated for clinical performance by Mater Pathology. Inner or nested amplicons were approximately 70 to 90 base pairs (bp) and the outer amplicons were up to 150 bp. All primers spanned intron-exon boundaries and were preferentially designed toward the 3' end.

In this reverse transcription and amplicon enrichment stage, reactions were performed using 20 ul volumes consisting of 10 ul of Step 1 mastermix, 8 ul of molecular biology grade (diethylpyrocarbonate) water, and 2 ul of RNA template (minimally 10 ug). Final target amplification process of multiplexed amplicons (Step 2), utilised involved preparation of the following PCR mix: 10 ul of the Step 1 reaction, was added to 550 ul of DEPC water and 560 ul of Mastermix. Twenty microliter aliquots of this Step 2 PCR mix were then added to the corresponding gene disk containing the lyophilized inner or nested primers. Amplification was performed on the RG6000 (Qiagen) thermal cycler.

Gene expression levels, expressed as "relative fold change", were determined using a method comparable to the 2^-ΔΔCt ^method described by Pfaffl [16], where the point of peak cycling acceleration was extrapolated to represent maximum fold change. JAG1, FUK and PRDM8 were used as normalisation control genes in the calculation of gene expression fold changes. Five negative controls (using DEPC water as a template), and five positive controls (using commercially available Universal RNA as a template) were included as references.

### Bioinformatics and statistical analysis of data

Four multi-feature classifiers were used to separate HC versus Mixed Inflammation (MI included both PS and sepsis groups), as well as PS Vs sepsis using the HGU133 Plus 2.0 gene expression data. These classification techniques include Recursive Partitioning, Figueiredo's method, the "least absolute shrinkage and selection operator" or LASSO, and Logistic Regression on Principal Components. In particular, the first three of these four classifiers were applied as they have the capacity to identify small subsets of biomarkers using a high 'throughput' platform. Individual genes were examined using an empirical Bayes adjusted linear model [17], with *P-*values adjusted for multiple comparisons using Holm's method [18].

In the absence of a formal 'validation set' of samples in which to apply the algorithm created from the 'training set' of data, the Leave-One-Out Cross Validation technique was used. To estimate the error rate using this technique a sample from the original set was removed, the method rebuilt using the same procedure as before (including re-running of the pre-selection step), and this model used to predict the 'left out' sample. This is repeated so that each sample is sequentially left out, and the error rate computed in its absence.

To further validate the microarray data, the SeptiCyte Lab signature was applied to all currently available Gene Expression Omnibus (GEO) HGU133 Plus 2.0 GeneChip data derived from human whole blood leucoctye-based gene expression studies. The GEO database is an international publicly available archive of GeneChip data that can be used in research and development, for interrogation of proprietary gene signatures to support assessments of diagnostic utility. Following a review of this public database, an additional set of controls (*n *= 164) with no known systemic inflammatory or immunological conditions were added to the microarray study cohort.

For the MT-PCR analyses, a panel of maximally 42 genes (also known as SeptiCyte Lab) was used to generate a diagnostic classifier using a LogitBoost machine learning algorithm [19]. The data were randomly partitioned into a training and validation set. The LogitBoost algorithm was used on the training set, to generate a classifier. The classifier was then applied to the validation set. Posterior probabilities of each condition were obtained for the validation set. These posterior probabilities were used as a diagnostic index, and the diagnostic performance was assessed using a receiver operator characteristics (ROC) curve [20,21]. After the initial area under the ROC curve was calculated the procedure was repeated, such that the data were randomly partitioned 500 times into training and validation sets, and the ROC area calculated for each random partition. The procedure was also conducted to include a random permutation of group labels in the training and validation sets. The distribution of ROC areas was calculated under this permutation scheme.

It should be noted that no tabulations of sensitivity or specificity have been provided, since these parameters have meaning only in the context of a well-defined clinical population, where the population has been sampled more extensively than that described in this study. Furthermore, sensitivity and specificity may always be traded, that is, specificity may be increased by reducing sensitivity or vice versa. Thus, meaningful claims about sensitivity and specificity can only be made in late-stage clinical research, when the diagnostic thresholds have been established using large cohorts of blinded samples.

## Results and discussion

**Figure 1:**
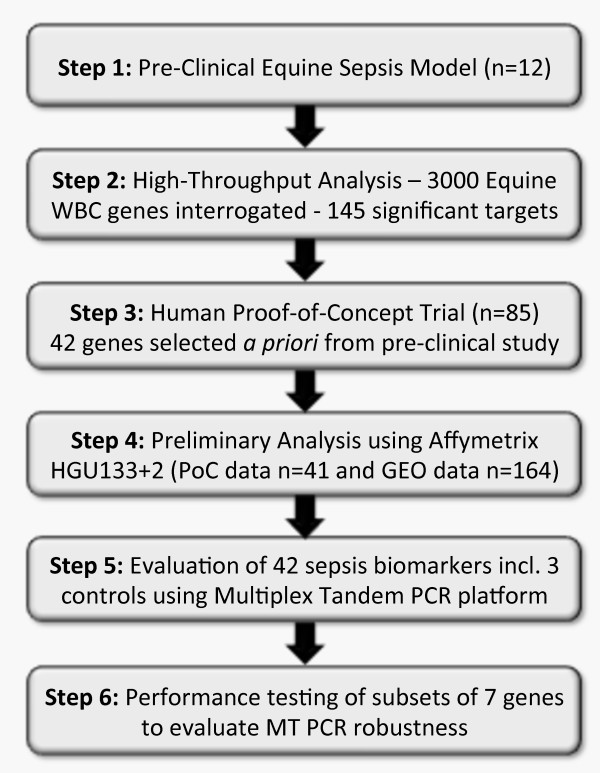
**A schematic of SeptiCyte lab development**. Summary of the development pathway of the SeptiCyte Lab test from high throughput pre-clinical gene expression interrogation in an equine sepsis model through to clinical evaluation on a more dynamic platform better suited to the assessment of smaller biomarker panels.

Pre-clinical research using a novel equine model of sepsis was designed to examine the innate and adaptive immune responses. In brief, endotoxemia and sepsis was induced in a cohort of foals (*n *= 12) after animals were administered a high carbohydrate diet for 48 hours causing damage to the lining of the intestine and allowing live and dead bacteria to invade the bloodstream, resulting in bacteremia and multiple organ dysfunction. Given recent findings reported by Wade and colleagues [22], where it was demonstrated that 53% of equine chromosomes show conserved synteny to a single human chromosome, this suggests that the horse genome has strong concordance with that of the human and, validates this model as an appropriate one for clinical research and development. Furthermore, based on these pre-clinical findings, a complete clinical research program was developed in order to establish the utility of the biomarkers for the early diagnosis of sepsis (Figure 1).

**Figure 2:**
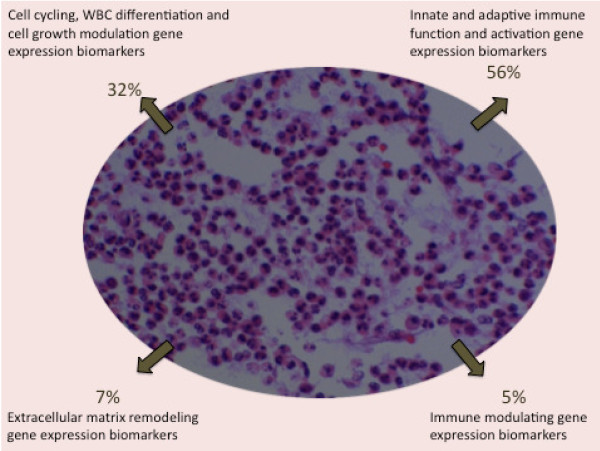
**Composition of gene expression biomarkers in the SeptiCyte Lab test**. Based on longitudinal sampling in pre-clinical trials using an equine sepsis model, molecular biomarkers related to genes directly involved in innate and early adaptive immune function, cell cycling, differentiation, extracellular remodelling, as well as immune modulation.

From these studies, 145 human white blood cell (WBC) genes associated with acute infection and inflammation were identified as being abnormally expressed both in the equine and human contexts. Following, a panel of 42 genes were identified *a priori *using pre-clinical research outcomes from the pre-clinical results. This panel of biomarkers has been primarily linked with innate and early adaptive immune function/activation pathways, including those governing cell cycling and growth, WBC differentiation, extracellular matrix remodelling, and immune modulation (Figure 2). Based on the physiological response to infection, that is, monocytosis, neutrophil activation, monocyte-macrophage differentiation, extravasation, as well as cell-mediated responses, these patented markers appear to characterise a large cross-section of early immune behaviour. We, therefore, developed a clinical research program to validate this novel biomarker panel to determine its efficacy as a diagnostic test for the separation of sepsis within an inflammatory milieu.

A total of 85 participants were enrolled in this study to be assessed for sepsis status using a gene expression biomarker test (SeptiCyte Lab). The baseline characteristics between the PS and sepsis cohorts were comparable in terms of age, sex and ethnic background; however, while demographic variables were well matched between the healthy control (HC) and in-patient groups, the HC were significantly younger (Table 1).

**Table 1:** Baseline characteristics of the study population

Characteristic	HC (*N *= 20)	Sepsis (*N *= 27)	PS (*N *= 38)	*P*-value*
**Age-yr**				
Mean	38	60	68	NS
Range	21 to 60	38 to 82	51 to 86	NS
**Male sex -- %**	50	55	64	NS
**Caucasian -- %**	100	94	100	NS

* Statistical comparisons made between Sepsis and PS

Of the surgical in-patients enrolled into this study, 19 had major open cardiac surgery that included CABG, pericardiectomy, valve replacement and atrial septal defect closure procedures. A further 14 participants had major thoracic procedures that included pleurodesis, lung wedge resection with and without lung volume reduction, lung lobectomy and pulmonary decortication. Lastly, five participants underwent major open vascular and neurosurgery that included AAA repair, aortic, iliac, femoral and femoral-popliteal endarterecetomy procedures, as well as stereotactic removal of a cerebral lesion, respectively. Based on routine clinical catheter tip culture monitoring, none of these participants had any evidence of infection during, or after their participation in this trial; however, all of these surgical in-patients were minimally on broad-spectrum prophylactic antibiotic therapy at the time of the study blood collection.

Of the 27 patients recruited into this trial who met the criteria for sepsis, and who had evidence of systemic infection, 14 had microbiological findings consistent with a gram-positive infection, 11 had findings consistent with a gram-negative infection, and 2 had findings of both gram-negative and gram-positive infections. As this was an observational clinical study, all sepsis participants were treated as per clinical guidelines regardless of SeptiCyte Lab results. On average, the sepsis participants' stayed in the ICU for five days.

**Figure 3:**
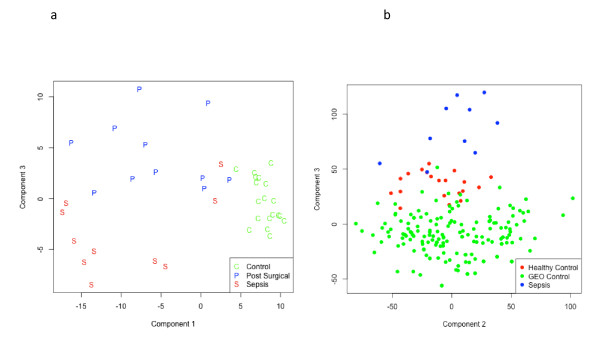
**Principal Component Analysis for preliminary HGU133 Plus 2.0 array studies**. A strong separation between HC (referred to as "Control") and MI groups and a moderate separation between PS and sepsis participants was noted (**A**), where all but one sepsis sample is below the PS threshold. Moreover, when GEO control samples were included in these analyses, control gene expression profiles were largely independent of the sepsis group (**B**).

In preliminary microarray investigations, HGU133 Plus 2.0 GeneChips demonstrated that signatures for HC, PS and sepsis were well separated when visualised using a Principal Component Analysis (Figure 3A). The area under the curve (AUC) for the ROC curve, using the HC, PS and sepsis data sets was greater than 95% for the detection of sepsis. To evaluate the robustness of this gene expression signature, GEO control samples were compared with gene expression data from the sepsis and HC cohorts. In Figure 3B a strong separation was observed between all 'controls' (GEO and HC) when compared with the sepsis cohort, thus generating a specificity of greater than 99% using ROC curve analyses.

The objective of the GEO data exploration was to investigate whether or not the diagnostic signature is robust to inter-laboratory variation. There is limited evidence that the signature is robust, but inter-laboratory variation in positive sepsis samples would have to be evaluated before this can be proven. Importantly, the high specificity for the GEO data must be interpreted with caution given that no sepsis data from the GEO database were included. Whilst the GEO database does contain gene expression information from sepsis trials, the clinical status of the participant cohorts appeared to be ambiguous based on Inclusion and Exclusion Criteria and for this reason, it was not included in these analyses. At this stage it may be the case that the very high specificity is adversely affecting sensitivity. However, it is encouraging that a classifier developed in one laboratory does not result in a large number of false positives when applied to samples from a large number of laboratories. This provides some evidence that the difference between sepsis and control patients from this trial is greater than the inter-laboratory variation in the gene expression data. Had the difference between control and sepsis patients from the current trial been small with respect to between laboratory variations in 'control' gene expression, it is likely to have resulted in the GEO data decreasing the sensitivity. In this sense, the GEO data provide preliminary evidence that the sensitivity of the test is robust to inter-laboratory variation.

It is routine practice in research and development that microarray studies be employed in preliminary investigations, particularly in molecular oncology, in order to identify statistically relevant panels in which to re-evaluate and further refine in the clinical setting [23-25]. Given that these analyses were not restricted to inflammatory biomarkers, these results provided a strong rationale for further clinical research.

When the *a priori *set of 145 biomarkers was compared with all gene expression change from the Affymetrix Genechip data they were strongly representative of widespread change and, had the greatest capacity to differentiate within the MI cohort. Thus, based on preliminary outcomes, a further subset of 42 biomarkers including three control/normalisation genes were tested in a larger cohort of critical care patients, to ascertain the clinical utility of this sepsis test using the more efficient quantitative real time PCR platform.

**Figure 4:**
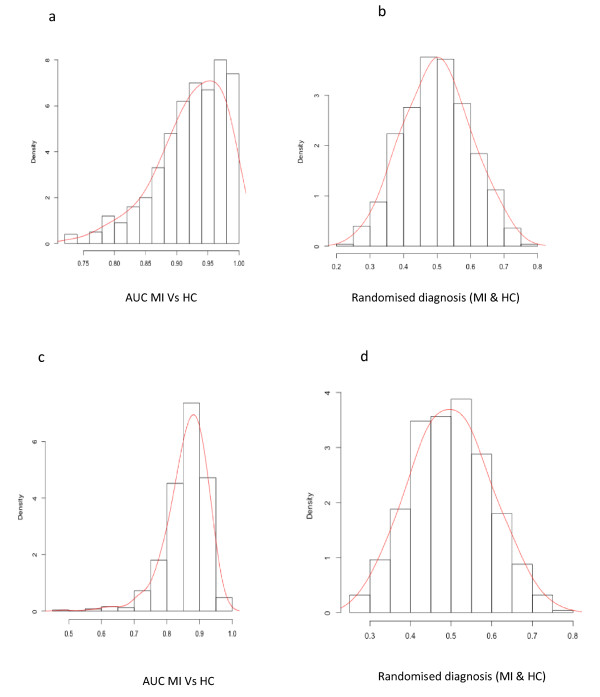
**AUC ROC permutation comparisons and distribution graphs between mixed inflammation and Healthy Control cohorts**. **A **and **B **display results for the AUC ROC following 500 iterations using the full gene set and distribution results when cohort labels are removed, respectively. In **C **and **D **this analysis was repeated using a diagnostic signature that is operating with a set of seven genes, where minimal change in performance was noted.

Following MT-PCR data extrapolation, comparisons were first made between HC and generalised systemic inflammation and wounding (MI). Findings from these analyses indicate, based on area under the curve (AUC) for ROC curve, that the ability to accurately detect severe systemic inflammation was approximately 92% using the full gene panel (Table 2). Based on further posterior probability analyses that were conducted using algorithms for 42 and 7 gene expression biomarkers (Figure 4A, C), this result was determined to be statistically significant (*P *< 0.002) in a permutation test. Furthermore, to demonstrate that there was no underlying bias in the patient population, the HC and MI cohorts had classifiers removed and were randomly mixed during posterior probability analyses, indicating that diagnostic performance (by chance) was approximately 0.5 (Figure 4B, D).

**Table 2:** AUC ROC results comparing diagnostic performance between control and various clinical cohorts

Comparison	Biomarker Set	Mean	SD‡	*P*-value*
MI Vs HC	42	0.921	0.0568	< 0.002
MI Vs HC	7	0.862	0.0640	< 0.002
Sepsis Vs HC	42	0.938	0.0557	< 0.002
Sepsis Vs HC	7	0.910	0.0479	< 0.002
PS Vs HC	42	0.891	0.0687	< 0.002
PS Vs HC	7	0.833	0.0661	< 0.002
Sepsis Vs PS	42	0.921	0.0568	< 0.002
Sepsis Vs PS	7	0.862	0.0640	< 0.002

‡ Standard Deviation; *Statistical significance is assumed when *P *< 0.05. Based on 500 random permutations the minimum possible *P*-value is < 0.002

§ HC -- Healthy Control; MI -- Mixed Inflammation; PS-Post Surgical

**Figure 5:**
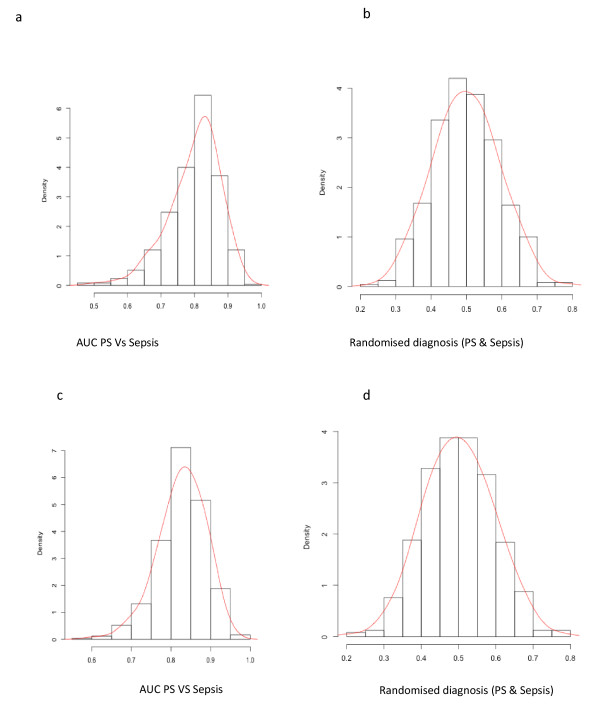
**AUC ROC permutation comparisons and distribution graphs between PS and Sepsis cohorts**. Permutation analyses were conducted using 500 iterations, where the data was randomly split into training and validation sets to evaluate diagnostic performance. Average AUC ROC for gene sets of 42 and 7 were determined to be 0.92 ± 0.0586 and 0.862 ± 0.0640, respectively. When labels associated with cohort status were removed, the AUC distribution was on average 0.498 indicating that there was no inherent bias in this methodology.

Secondly, the mixed inflammatory cohort was split into PS and sepsis, where AUC ROC permutation results for the full gene set (*n *= 42) and a sub-set (*n *= 7) demonstrate the ability to detect sepsis was between 86 and 92% (*P *< 0.002), as displayed in Figure 5A, C. Again, when participant classifiers were removed for the purpose of posterior probability analyses, random diagnostic performance was on average 0.5 (Figure 5B, D).

Interestingly, when small sets of gene expression markers are used, the performance of the algorithm to accurately detect sepsis remained high. In fact, several sets of seven biomarkers were evaluated, and performance remained high for each set, suggesting that there is substantial redundancy in the 42-biomarker set. Despite this redundancy, the SeptiCyte Lab will continue to include all 42 biomarkers for future studies designed to evaluate sepsis status in patient populations from different settings such as: in neonatology and paediatrics settings, patients who are immune-compromised following chemotherapy, as well as in mixed ethnic populations. The larger biomarker set provides a higher chance of success for these more diverse populations.

Quantitative real time PCR, also referred to as the MT-PCR method in this paper, is a fast and efficient platform on which to conduct gene expression studies, and an ideal platform for use in a tertiary care clinical pathology unit. Given the current level of automation, and the requirement for as little as 10 ng of RNA (< 1 PAXgene or EDTA blood sample), test results can be reported back to the referring clinical team within three hours. Hence, there is the capacity to substantively improve early clinical management plans when considering it may take between 48 and 72 hours for blood culture results to become available, a limitation that is further exacerbated by the failure to grow an organism in a high percentage of blood cultures taken from patients who have been deemed to be extremely likely to have sepsis on clinical grounds.

To date, the application of such a highly multiplexed gene expression test for the early detection of sepsis is unique; however, there are similar diagnostic products on the market for other immune-related conditions in the field of heart-lung transplantation [26]. Importantly, multi-marker diagnostics may provide guidance where there is nonlinearity of data, a situation commonly associated with complex disease states.

In terms of limitations, this test currently has the capacity to distinguish between normal individuals (healthy controls) versus those with systemic inflammation of unknown aetiology (MI), as well as PS inflammation (surrogate SIRS) and sepsis (confirmed bacteraemia). However, as indicated there are a number of 'high risk' populations that develop infection due to immunomodulating medications used following transplantation, chemotherapy regimens and management of autoimmune diseases. To use this test efficaciously in such complex cases would require baseline gene signatures for these populations. Further studies are underway to derive these.

## Conclusions

In summary, these findings demonstrate that this novel gene expression biomarker test (SeptiCyte Lab) has the capability, based on diagnostic performance outcomes, to accurately detect early evidence of sepsis well before the availability of microbiology results. Current clinical research will further investigate the efficacy of this assay to provide guidance on the host immune response via the monitoring of severe sepsis patients, as well as to provide diagnostic information on other 'at risk' groups such as post-operatively following major surgery and patients with chronic immune incompetence.

## Key messages

• Using results from an equine sepsis model, 42 genes were selected *a priori *to test a novel gene expression panel using biomarkers associated with innate and early adaptive immune function, cell cycling and growth, WBC differentiation, extracellular remodelling and immune modulation.

• Preliminary microarray studies using a PCA suggests that the detection of sepsis in a group of HC, PS and sepsis patients was > 95%, a finding that increased to > 99% when further GEO control data were included in the analysis.

• MT-PCR data analysed using multi-classifier techniques, demonstrated that the average area under the ROC curve for sepsis detection was approximately 92% (*P *< 0.02), in a mixed inflammation group (post-surgical and sepsis patients).

• Applying a sub-set of gene expression markers (*n *= 7), the average area under the ROC curve for sepsis prediction in a mixed inflammation group was approximately 86% (*P *< 0.02).

• Based on overall outcomes, these findings suggest that this sepsis gene expression test is robust; ongoing studies will determine whether it performs equally well when applied to different patient populations and ethnic groups.

## Abbreviations

AUC: area under the curve; DAI: dependent activator of interferon; DAMPs: damage (or danger)-associated molecular patterns; GEO: Gene Expression Omnibus; HC: healthy control (participant); HRECs: Human Research Ethics Committees; IRBs: institutional review boards; LASSO: least absolute shrinkage and selection operator; MHS: Mater Health Services; MI: mixed inflammatory (group); MT-PCR: multiplex tandem-polymerase chain reaction; PS: post-surgical (participant); ROC: receiver operator characteristics; SIRS: Systemic Inflammatory Response Syndrome; WBC: white blood cells

## Competing interests

### Financial competing interests

AS is a research consultant and clinical research manager for Athlomics P/L. RAB is the Chief Executive Officer Athlomics P/L and also holds shares in the company, as well as being a founder. RBB, MT and DV are founders of Athlomics P/L and also hold shares within the company. Mater Pathology (employer of DV, GP and TN) received remuneration for expenses incurred during the conduct of gene expression studies. Site investigators (JL and RP) have been reimbursed for the cost of patient recruitment and data collection. GS was remunerated as a statistical consultant to conduct bioinformatics. Mater Health Services (MHS) owns 2% equity in Athlomics P/L.

A US provisional patent application (61/360,430) was filed on the 30 June 2010 by Athlomics Pty Ltd and covers all intellectual property relating to the scientific concepts and findings presented within the manuscript.

### Non-financial competing interests

The authors declare that they have no competing interests.

## Authors' contributions

RAB, RBB, MT, AS and DV conceived the study and had input into the study design, analysis of data and written communication of the findings (manuscript). JL, BT, AM and RP were responsible for the coordination and conduct of the data collection as well as critical review of the manuscript. MT and GS were responsible for conducting the bioinformatics and statistical analyses. GP and TN performed the microarray studies. All authors have read and approved the manuscript.
